# Features and Associated Comorbidities of Laryngomalacia in Saudi Arabia

**DOI:** 10.7759/cureus.47432

**Published:** 2023-10-21

**Authors:** Omar Alanzi, Moath Al-faleh, Hussain Alsheef

**Affiliations:** 1 Otorhinolaryngology, King Fahad Specialist Hospital, Dammam, SAU; 2 Pediatric Otorhinolaryngology, Maternity and Children Hospital, Dammam, SAU

**Keywords:** noisy breathing, respiratory distress, neonate, stridor, laryngomalacia

## Abstract

Laryngomalacia (LM) is defined as the collapse of supraglottic structures during inspiration, resulting in intermittent airflow impedance and associated stridor. LM is the most prevalent cause of congenital stridor in newborns. The aim of this study is to determine the features and associated comorbidities of LM in Saudi Arabia and to delineate the diagnostic and therapeutic measures used, based on the severity of the case and related comorbidities. This is a three-year retrospective study of children diagnosed with LM and treated in the pediatric otorhinolaryngology outpatient clinic at Maternity and Children Hospital, Dammam, Saudi Arabia, between January 2018 and January 2022. The inclusion criteria were patients with signs and symptoms of LM who are younger than 14 years old. The diagnosis of LM was based on clinical evaluation and confirmed by nasopharyngolaryngoscopy in awake patients and/or direct laryngoscopy and scoping under general anesthesia with spontaneous ventilation for dynamic evaluation. The Olney classification was used for the morphological classification of LM. The exclusion criteria were patients lost to follow-ups. Follow-up duration was two years minimum. A total of 52 patients were included in the study. Among the participants, females accounted for 71% and males accounted for 29% of cases. Our results were in accordance with the relevant literature, except for the higher prevalence of LM in full-term neonates who were found to account for 69.2% of the cases. Understanding the patterns and characteristics of breathing may help clinicians distinguish the noisy breathing of LM from other illnesses because infants are frequently misdiagnosed with these conditions.

## Introduction

Laryngomalacia (LM) is defined as the collapse of supraglottic structures during inspiration, resulting in intermittent airflow impedance and associated stridor. A big percentage (45-75%) of infants with congenital stridor have LM, which is the most prevalent cause of stridor in newborns [1]. Parents and other caregivers may find stridor to be overwhelming. Inspiratory stridor, which is a symptom of LM, frequently gets worse with feeding, crying, lying down, and exertion. The signs and symptoms start soon after delivery or in the first few weeks of life, peak between six and eight months, and usually resolve between 12 and 24 months of age, but it is vital to understand that not all cases of LM have a benign course [1,2].

The most common feeding-related symptoms are regurgitation, emesis, coughing, choking, and feeding difficulties. Due to their airway blockage, infants with LM may find it challenging to coordinate the suck-swallow breath sequence required for feeding [3]. Due to the higher metabolic demand of synchronizing breathing and eating while overcoming the obstruction, weight loss and failure to thrive may occur. Respiratory distress signs and symptoms and obstructive sleep apnea are other less frequent but alarming flags. If not identified and treated, chronic hypoxia from airway blockage can result in pulmonary hypertension [2,3,4]. Understanding the patterns and characteristics of breathing may help clinicians distinguish the noisy breathing of LM from other illnesses, such as croup, subglottic stenosis, vocal cord paralysis, tracheomalacia, and hyperactive airway, because infants are frequently misdiagnosed with these conditions [4].

The aim of this study is to determine the features and associated comorbidities of LM in Saudi Arabia and to delineate the diagnostic and therapeutic measures used based on the severity of the case and associated comorbidities. To the best of our knowledge, this is the first study of its kind to be conducted in Saudi Arabia.

## Materials and methods

This is a three-year retrospective study of children diagnosed with LM and treated in the pediatric otorhinolaryngology outpatient clinic at Maternity and Children Hospital, Dammam, Saudi Arabia, between January 2018 and January 2022. 

Patients with signs and symptoms of LM who are younger than 14 years old were included. The diagnosis of LM was based on clinical evaluation and confirmed by nasopharyngolaryngoscopy in awake patients and/or direct laryngoscopy and scoping under general anesthesia with spontaneous ventilation for dynamic evaluation. The Olney classification was used for the morphological classification of LM. Meanwhile, patients lost to follow-ups were excluded from the study. The follow-up duration was two years minimum.

Our management approaches when treating LM were as follows: observe the patient first with referral to other specialty when needed [u1]. Comorbidities were diagnosed and managed by a specialized physician or in the Maternity and Children Hospital, Dammam, Saudi Arabia. If the condition worsens or shows no signs of improvement, medical treatment and finally surgical management were implemented. The primary surgical treatment is supraglottoplasty. Tracheostomy is reserved for cases of surgical failure or in infants with numerous medical comorbidities. Clinical and demographic data along with comorbidities, associated synchronous airway lesions (SALs), and morphological type of LM were retrieved from electronic medical records of children diagnosed with LM. All data were analyzed using the IBM SPSS Statistics for Windows, version 25 (released 2017; IBM Corp., Armonk, New York, United States). The study's Institutional Review Board National Registration Number is H-05-D-114, and its Institutional Review Board number is PEDI-2023-0015.

## Results

**Figure 1:**
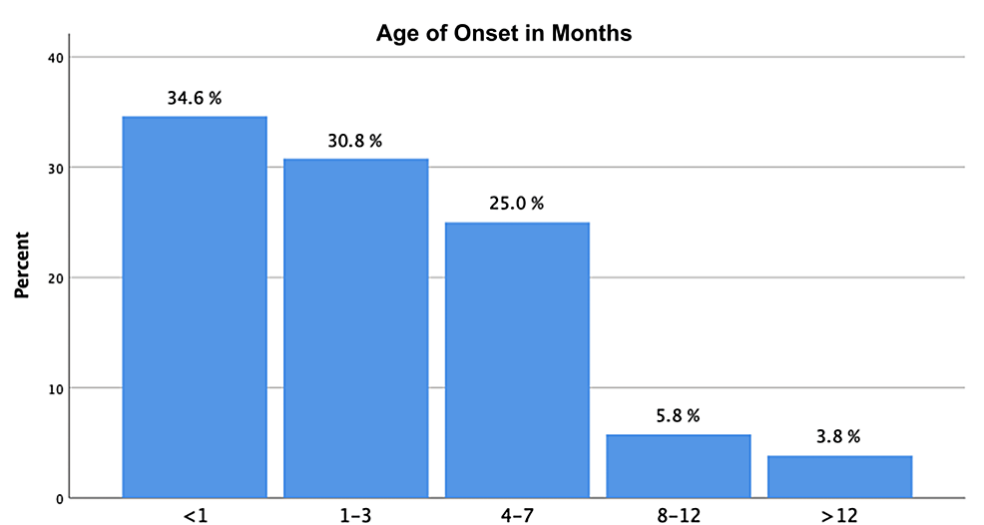
Age of onset of laryngomalacia symptoms among the participants.

A total of 52 patients were included in the study. Among the participants, females accounted for 71%, and males accounted for 29% of cases. Full-term children were 69.2%. The mean age of the participant is 21 months. The age of onset is well demonstrated in Figure 1, showing that 65.4% of the cases presented in the first three months of life. Based on the Olney grading system of LM, in our study, we found that mild and moderate LM are the predominant types, respectively (Table 1). SALs were diagnosed in 11 (21.2%) of the patients, and more were found in the neonatal age group, i.e., six (35%) of the total cases. All patients with SALs were diagnosed before the eighth month of life.

**Table 1:** Distribution of LM types based on the Olney grading system.

Grade	Number of patients	%
Grade I (Mild)	22	42.3%
Grade II (Moderate)	28	53.8%
Grade III (Severe)	2	3.8%

**Figure 2:**
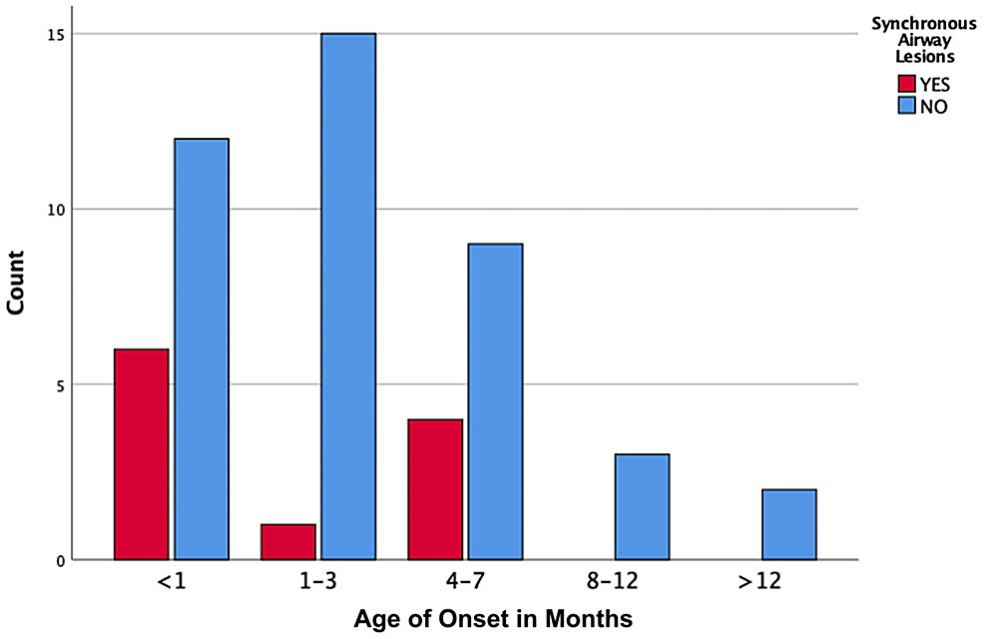
Prevalence of synchronous airway lesions by age groups.

**Figure 3:**
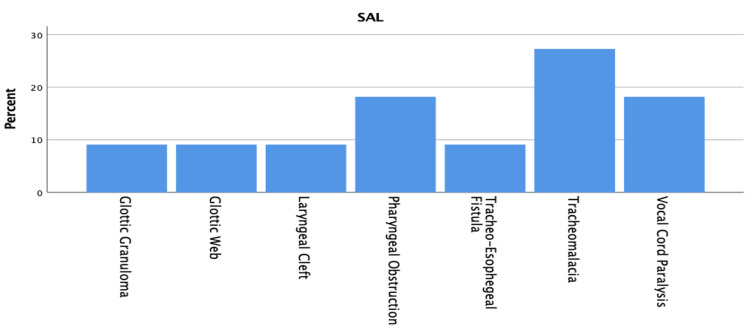
Distribution of synchronous airway lesions.

Figure 2 demonstrates the distribution of SALs by age group, and Figure 3 demonstrates the distribution of SALs found among the patients. Respiratory support in the form of endotracheal intubation was needed in 10 (19.2%) of the cases, 72% of which were associated with comorbidities and 36% associated with SALs.

Table 2 demonstrates the associated SALs in a patient who required endotracheal intubation. Table 3 shows the distribution of medical comorbidities in our study, which reveals that neurological disorders and gastroesophageal reflux disease (GERD)/laryngopharyngeal reflux (LPR) are the most common associated comorbidities. Surgical interventions were tailored based on the patients' conditions. Out of the 52 patients, a total of eight (15%) patients underwent surgical interventions because of significant respiratory symptoms and/or failure to thrive caused by SALs. The remaining patients had significant respiratory and nutritional improvement without surgery. 

**Table 2:** Associated synchronous airway lesions in a patient who required endotracheal Intubation.

Synchronous airway lesion	Endotracheal Intubation	Total
None	7	41
Glottic web	1	1
Tracheomalacia	1	3
Pharyngeal obstruction	0	2
Tracheoesophageal fistula	0	1
Laryngeal cleft	0	1
Vocal cord paralysis	0	2

**Table 3:** Distribution of medical comorbidities.

Comorbidity	N
Neurological	12
Gastroesophageal reflux disease (GERD)/laryngopharyngeal (LPR)	11
Cardiac	9
Down syndrome	3
Pulmonary	3
Metabolic	2
Congenital anomalies	2
Intrauterine growth restriction	1
Renal	1
Tracheoesophageal fistula	1

## Discussion

Gastroesophageal reflux disease (GERD) or laryngopharyngeal reflux (LPR), neurological disorders, congenital heart disease, congenital malformations/syndromes, or the development of an SAL are medical comorbidities linked to LM. They are more frequently discovered in people who have moderate and severe illness [3-5]. In addition to LM, 25-50% of patients with GERD or LPR will also have another comorbidity [6-9].

Laryngopharyngeal reflux

With an incidence of 65-100%, GERD and LPR are the most frequent medical comorbidities seen in babies with LM [4-8]. In the present study, GERD and LPR were present in 21% of the cases, which is significantly lower than what has been reported in the literature. Differences in the methods used to reach this diagnosis may account for the marked discrepancies in the incidence of GERD and LPR. In the present study, the diagnosis was based on clinical and endoscopic findings, which is a rather objective method with unclear diagnostic criteria.

Frequent LPR events cause inflammation and edema in the tissues of the upper airway, which can worsen airway obstruction and compromise it [4-8]. This can result in a vicious cycle of reflux and airway obstruction until one or both of them are resolved. The collapse of the supra-arytenoid into the glottic inlet as seen in form of LM is partially caused by edematous alterations of the larynx caused by LPR [10,11,12]. The coughing, choking, feeding, and aspiration symptoms that newborns with moderate to severe LM have are likely caused by the LPR impact on laryngeal feeling, which can cause issues with airway protection and swallowing [3,9,11,12]. Improvements in laryngeal feeling and swallowing efficiency have been linked to anti-reflux medications [3,11]. Treatment with anti-reflux medications showed improvement in the symptoms and shortening the natural course of the disease [3].

Synchronous airway lesions

The prevalence of SALs varies from 7.5% to 64%; the wide range is probably explained by the method of diagnosis and the lack of consensus on the examination of choice and the diagnostic criteria. Examinations under anesthesia using a flexible or rigid approach has become the norm. For dynamic lesions, a flexible methodology is preferable to a rigid technique, and vice versa for fixed lesions [12-16]. Diagnoses of fixed lesions are difficult to make if flexible endoscopy alone is employed to evaluate SALs [3]. In our study, SALs were diagnosed in 11 (21.2%) patients and found more in the neonatal age group, where they accounted for six (35%) of the total cases. Studies suggest that SALs are less expected if other medical comorbidities are present or among preterm neonates; however, in patients with severe obstructive symptoms, SALs must be ruled out or treated if present [5,14-17]. Newborns with SALs are 4.8 times more likely to need surgery than infants with mild or moderate illness [14]. Our results are in accordance with the relevant literature both in terms of prevalence and age group distribution. In our study, tracheomalacia followed by vocal fold paralysis is the most frequent SAL.

Cardiac disorders

Symptoms of LM, namely, cyanosis, apnea, and stridor, may coexist with congenital heart disease. The two conditions may in some cases deteriorate one another in terms of symptom severity and clinical stage. Congenital heart disease is far more common in people with moderate and severe LM than it is in people with mild LM. In the literature, it is estimated that 10% of babies with LM have cardiovascular illness documented as a comorbidity [3,16,17]. In our study, cardiac disorders were present in 20% of the patients. The prevalence of cardiac disorders in this study is higher than that in the relevant literature, probably because our data come from a tertiary hospital, with both cardiology and otolaryngology services. The consequences of cardiac illness, particularly hypoxia, are assumed to be the main reason to perform surgery. Supraglottoplasty may reduce airway obstruction and lessen the severity of hypoxia, but it would not cure heart disease if it is not eventually treated. The effects of intervention on cyanosis are negligible. Compared to infants without comorbidity, infants with a heart disease require tracheostomy at a much greater rate [16-18].

Neurological disorders

With a frequency of 8-45% in the literature, neurologic illness is the second most often documented medical comorbidity [3,8,16]. It is also one of the most common indications for surgical treatments, since 30-45% of newborns with neurological illness need surgical intervention [3,13,18]. In our study, neurological disorders were considered the most common comorbidities, with a prevalence of 27%, as shown in Table 2. In the presence of neurological disorders, 60% of patients who undergo supraglottoplasty will need a tracheostomy, and up to 70% will require revision surgery [18]. In any infant with severe LM, aspiration, or hypotonia, the presence of a neurological disorder should be ruled out before proceeding to supraglottoplasty. The same applies if the patient continues having persistent feeding issues, aspiration, or airway symptoms after surgical management [17,18]. It is unclear exactly how coexisting neurologic disorders and LM relate to one another.

Congenital and genetic disorders** ** 

Down syndrome seems to be the genetic condition that is most frequently associated with respiratory symptoms in patients having LM [19-21]. In these cases, the disease responds well to intensive acid suppression therapy and supraglottoplasty, provided that there is no concurrent cardiac disease or neurologic disease [20-22]. The retrodisplacement of the tongue base compressing on the epiglottis, as well as supra-arytenoid tissue redundancy and short aryepiglottic folds, further deteriorate the severity of LM in patients with disorders linked to micrognathia, such as CHARGE Association and Pierre Robin sequence. The majority of these patients with significant airway obstruction and LM will need a tracheostomy until they outgrow the micrognathia or undergo surgical surgery to repair it [22,23]. Supraglottoplasty or epiglottic suspension treatments are typically of low success rate in the presence of micrognathia. When severe LM necessitates intervention, a syndrome or anomaly should not prevent supraglottoplasty if micrognathia is absent. However, there may be a higher likelihood of failure and tracheostomy placement [23-26].

Medical interventions

The majority of cases with LM (70-90%) are mild and manifest as episodic, isolated stridor without changes due to crying or coughing, dyspnea, or swallowing issues. These minor types have no effect on the infant's growth; the physician merely needs to monitor the child to look for any signs of severity. Poor weight gain, dyspnea, episodes of respiratory distress, obstructive sleep apnea, and feeding issues are indicators of severity [1,2]. Lifestyle modifications, such as thickening milk, maintaining posture after feeding, and elevating the head of the bed, are the first step of management. Along with lifestyle modifications, proton pump inhibitors and/or H2 histamine antagonists are used to break the cycle as mentioned earlier and therefore should be implemented as dietary measures [3,9-13].

Surgical interventions

Despite the different classifications of LM, all authors consider how severe the symptoms of stridor are. Therefore, when LM is present along with one or more of the following symptoms, surgery should be recommended: dyspnea with persistent, severe intercostal or suprasternal retraction; episodes of respiratory distress; obstructive sleep apnea; episodes of suffocation while feeding; and poor weight gain [2,26,27]. Regarding surgical interventions in our study, it was tailored based on the patients' condition. Out of 52 patients, eight (15%) patients underwent surgical interventions because of significant respiratory symptoms and/or failure to thrive caused by SALs. The remaining patients had significant respiratory and nutritional improvement without surgery. According to the literature, the need of surgical intervention is 4.2-10%; our higher rate can be due to the relatively small sample size and the fact that severe, complex cases with comorbidities usually referred to a tertiary center, such as ours [28,29].

To the best of our knowledge, this study is the first to be conducted in a tertiary hospital population in the Eastern region, Saudi Arabia. With this study, we aim to improve the quality of care provided, we however acknowledge as a limitation of our study the relatively small sample size and the retrospective review of medical data. Further multicenter studies are needed. Our results were in accordance with the relevant literature, except for the higher prevalence of neurological and cardiac comorbidities, which may be attributed to the fact that our data come exclusively from a tertiary hospital. In the absence of comorbidities, mild cases of LM are expected to be followed in secondary care hospitals. Another difference between the results of the present study and the existing literature in other geographical populations is that 69.2% of the participants were full term, which is against what was thought that LM is more common in the pre-term population. Seventy percent of LM cases in our study presented within three months of life, with the early presentation of LM being related to SAL.

## Conclusions

Understanding the patterns and characteristics of breathing may help clinicians distinguish the noisy breathing of LM from other illnesses because infants are frequently misdiagnosed with these conditions. Patients with LM should undergo a full airway evaluation to recognize the presence of comorbidities that may lead to sub- optimal outcomes and to rule out SALs to provide comprehensive management of young infants who present with signs or symptoms concerning for LM. Children not improved with supraglottoplasty often have underlying neurologic or syndromic abnormalities and may require a tracheostomy.
